# New Therapeutic Approaches for Conjunctival Melanoma—What We Know So Far and Where Therapy Is Potentially Heading: Focus on Lymphatic Vessels and Dendritic Cells

**DOI:** 10.3390/ijms23031478

**Published:** 2022-01-27

**Authors:** Jennifer Peil, Felix Bock, Friedemann Kiefer, Rebecca Schmidt, Ludwig M. Heindl, Claus Cursiefen, Simona L. Schlereth

**Affiliations:** 1 Department of Ophthalmology, Faculty of Medicine and University Hospital Cologne, University of Cologne, 50937 Cologne, Germany; jennifer.peil@uk-koeln.de (J.P.); felix.bock@uk-koeln.de (F.B.); ludwig.heindl@uk-koeln.de (L.M.H.); claus.cursiefen@uk-koeln.de (C.C.); 2 Center for Molecular Medicine Cologne (CMMC), University of Cologne, 50937 Cologne, Germany; 3 European Institute for Molecular Imaging (EIMI), University of Münster, 48149 Münster, Germany; fkiefer@gwdg.de; 4 Department of Oral, Maxillofacial and Plastic Facial Surgery, Medical Faculty and University Hospital Düsseldorf, Heinrich Heine University, 40225 Düsseldorf, Germany; Buschr@hhu.de

**Keywords:** dendritic cells, conjunctival melanoma, cDC1, cDC2, pDCs, lymphatic, immunotherapy

## Abstract

Conjunctival melanoma (CM) accounts for 5% of all ocular melanomas and arises from malignantly transformed melanocytes in the conjunctival epithelium. Current therapies using surgical excision in combination with chemo- or cryotherapy still have high rates for recurrences and metastatic disease. Lately, novel signal transduction-targeted and immune checkpoint inhibitors like cytotoxic T-lymphocyte-associated protein 4 (CTLA-4) inhibitors, programmed cell death protein-1 (PD-1) receptor inhibitors, BRAF- or MEK-inhibitors for systemic treatment of melanoma have improved the outcome even for unresectable cutaneous melanoma, improving patient survival dramatically. The use of these therapies is now also recommended for CM; however, the immunological background of CM is barely known, underlining the need for research to better understand the immunological basics when treating CM patients with immunomodulatory therapies. Immune checkpoint inhibitors activate tumor defense by interrupting inhibitory interactions between tumor cells and T lymphocytes at the so-called checkpoints. The tumor cells exploit these inhibitory targets on T-cells that are usually used by dendritic cells (DCs). DCs are antigen-presenting cells at the forefront of immune response induction. They contribute to immune tolerance and immune defense but in the case of tumor development, immune tolerance is often prevalent. Enhancing the immune response via DCs, interfering with the lymphatic pathways during immune cell migration and tumor development and specifically targeting tumor cells is a major therapeutic opportunity for many tumor entities including CM. This review summarizes the current knowledge on the function of lymphatic vessels in tumor growth and immune cell transport and continues to compare DC subsets in CM with related melanomas, such as cutaneous melanoma and mucosal melanoma.

## 1. Introduction

Melanomas vary in their behavior, genetics, and response to therapies. It is, therefore, important to look at melanoma subtypes in a differentiated way. Currently, we are seeing significant therapeutic success for cutaneous melanoma with molecular inhibitors and immune checkpoint inhibitors. Success in ocular melanoma is limited because of the lack of therapeutic standards and clinical trials for conjunctival melanoma (CM) and, in general, too little is known about the immunology of ocular tumors and the tumor microenvironment. We, therefore, may miss ocular features if drugs tested only for cutaneous melanomas are uncritically applied to ocular melanoma patients. It is important not to consider or treat all melanomas the same. For example, another completely different ocular melanoma subtype is uveal melanoma. It originates from non-epithelial melanocytes and differs significantly compared to dermal, mucosal and conjunctival melanoma in terms of mutations, with mostly primary mutations located in *GNAQ* or *GNA11*, [1,2], which are not of importance in cutaneous melanoma or CM. Uveal melanoma metastasizes, primarily hematogenous, into the liver, different from CM and cutaneous melanoma and does respond less to immune checkpoint inhibitors (20–30%) [3], compared to metastatic cutaneous melanoma (40–60%) [4].

The overall mortality rate, e.g., for CM, is still high and is approximately 30% [5]. Therefore, the improvement of systemic or local therapies, especially for metastatic cases, is crucial. There are some particularities especially in the eye compared to the skin. Here, resection of larger tumors or the adherence to all resection margins may be associated with functional losses.

This review summarizes the similarities and differences of CM (summarized in Table 1) compared with its closest relatives, cutaneous melanoma and mucosal melanoma, and highlights new therapeutic approaches for the future for all three forms of melanoma. We address the epidemiology, clinic and etiology, and genetics of CM, cutaneous melanoma, and mucosal melanoma. Current and new therapeutic approaches will be discussed. The focus of potential new therapeutic approaches includes mainly dendritic cells (DCs) and lymphangiogenesis.

## 2. Conjunctival Melanoma

### 2.1. Epidemiology

Five percent of all ocular melanomas are CMs [5]. The incidence in the Caucasian population is 0.2–0.8 cases per million and year, with men and women being equally affected [22,23,24,25,26]. In relation to the surface area, the incidence of CM is equal to cutaneous melanoma [27]. The mean age of diagnosis is between 55–65 years [5,17,18]. Less than 1% of CM cases occur within the black population and the tumor is also very rare in Asians [22,28], but with an increasing incidence [29,30]. An increase in incidence is also seen in cutaneous melanoma, but not in uveal melanoma [31].

### 2.2. Clinic, Etiology and Risk Factors

**Figure 1:**
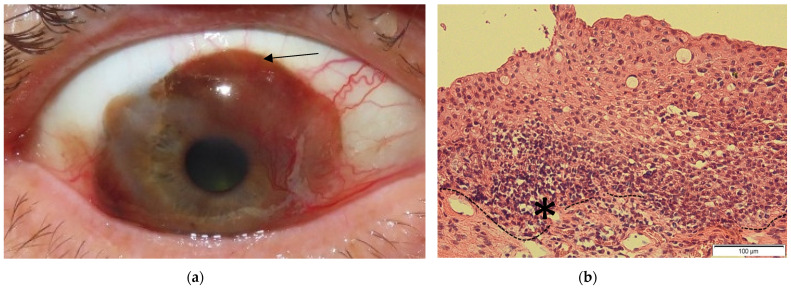
(**a**) Clinical picture of CM shows the pigmented tumor overgrowing the limbus and prominent feeder vessels (arrow); (**b**) Histological picture (Hematoxylin Eosin) of CM: The architecture of the epithelium is destroyed and the basal membrane (broken line) is disrupted by the tumor. Asterisk (*) marks an accumulation of atypical melanocytes characterized by pyknic and basophil cell bodies.

CM is an asymptomatic pigmented plaque, macule, or tumor often surrounded by feeder blood vessels [17,24,32]. CM appears on bulbar conjunctiva in 92% of cases, the temporal quadrant (63%), touches the limbus (61%) and can also appear in the palpebral and forniceal conjunctiva, plica semilunaris and caruncula [32] (Figure 1). CM arises from malignantly transformed melanocytes in the basal layer of the epithelium [5,33]. The risk factors of CM are not completely understood. For cutaneous melanoma, it is known that risk factors are, for example, family history, UV light exposure and genetic syndromes like xeroderma pigmentosum, Hodgkin lymphoma and familial melanoma syndromes [34,35,36,37,38]. In CM there are published case reports of patients with Xeroderma pigmentosum and CM [37,39]. The conjunctiva is the only mucous membrane exposed to UV radiation. This sunlight exposition is thought to be involved in the development of sun-exposed CM [40,41,42].

Several studies indicate that mucosal melanoma is not associated with human papilloma viruses, human herpes viruses and polyomaviruses [43,44,45,46]. It has been suggested that smoking is a risk factor for oral mucosal melanoma because oral pigmented lesions are more frequent among smokers [47]. Exposure to formaldehyde was suggested as risk factor for sinonasal mucosal melanoma [48,49]. Patients with HIV have a higher risk to develop anorectal mucosal melanoma [43]. Risk factors for vulvovaginal mucosal melanoma are potentially chronic inflammatory conditions, viral infections and chemical irritants [43]. CM is associated with conjunctival intraepithelial, melanocytic neoplasia (C-MIN, formally called primary acquired melanosis (PAM)) and nevi [5,50]. Especially in C-MIN with atypia, there is a high CM risk (53% to 75% of all cases) [17]. CM can further develop from a conjunctival nevus, which occurs in 5% of the cases, or can be formed de novo without any preceding lesion, which occurs in 18% to 30% of the cases [51,52,53].

### 2.3. Genetic Background

In CM, mutations in the mitogen-activated protein kinase (*MAPK*) and Phosphoinositide 3-kinases (PI3K)/protein kinase B (AKT)/mechanistic target of rapamycin (mTOR) pathway have been shown [54,55,56]. Mutations in the proto-oncogenes *NRAS, BRAF*, *KIT*, or the tumor suppressor gene neurofibromin 1 (*NF1)* lead to an increase in signal transduction from the cell membrane to the nucleus, which in turn activates differentiation, growth and survival [57,58,59]. Mutations in *NRAS*, a small GTPase, are found in approximately 20% of CM and in up to 30% of cutaneous and other mucosal melanomas [20,40,57,58,60,61,62]. *NRAS* activates *BRAF* a component of the MAPK pathway and PI3-K of the PI3-K/AKT/mTOR pathway [63,64]. Mutations in *BRAF* occur in 14–50% of CM cases. The most frequent *BRAF* mutation is the V600E mutation (80–90%), [62,65,66,67]. *BRAF* activates the mitogen-activated protein kinase (MEK) protein by phosphorylation, which leads to activation of the extracellular-signal regulated kinases (ERK) protein, leading to cell growth [59].

*BRAF*^V600E^ mutations are typically found in cutaneous melanoma areas without sun damage and develop from nevi [68,69], while atypical mutations in *BRAF* are found in sun exposed skin melanoma [70]. Compared to the number of *BRAF* mutations in cutaneous melanoma (32–60%) [20,56,71,72,73,74], it is relatively rare in other mucosal melanomas (3–10%) [20,75,76,77,78,79]. In CM, *BRAF* mutations are found in the sun-exposed epibulbar conjunctiva with a more than twofold frequency [68].

An *NF1* dysfunction occurs in 30% of CM, in 8–18% in mucosal melanoma and in 12–25% in cutaneous melanoma [20,80]. *NF1* is, as mentioned above, a tumor suppressor protein [81].

Mutations in the promoter of the telomerase reverse transcriptase (*TERT*) are found in 32–43% of CM [41,82]. *TERT* mutations are mainly found in UV exposed areas and correlate with a shorter metastatic free survival [82]. They are not detectable in conjunctival nevi or PAM without atypia [83]. *TERT* mutations have been seen in 85% of metastatic cutaneous melanoma tissues, and 33% of primary cutaneous melanomas [84], but are very rare in mucosal melanoma (8%) [85].

Mutations in the receptor tyrosine kinase *KIT* (CD117), a member of the PI3K/AKT/mTOR pathway, have been detected in 0–11% of CM cases [17,20,86]. *KIT* mutations are very rare in cutaneous melanoma (0–2%) and occur more often in mucosal melanoma (4–21% of cases) [20,21]. The mutational activation of *KIT* leads to ligand-independent auto-phosphorylation of intracellular tyrosine residues, resulting in downstream activation of PI3K [87]. *KIT* is crucial for the normal development of melanocytes acting as an important mitogen [88].

### 2.4. Prognosis

The total mortality of CM is around 30% [5]. The tumor can metastasize via lymphatic and hematogenous pathways and rapidly colonizes draining regional lymph nodes [17,52,89]. Distant metastases are seen in the liver, brain, skin, gastrointestinal tract and lungs [90,91]. The survival rate of CM is 74–86% after five years and 41–78% after 10 years [30,51,92,93,94]. The local recurrence rate of 36–62% is high [17,95]. This poor prognosis may improve in the future, due to novel immune checkpoint therapies, but except for case reports, prognostic data for larger cohorts are presently not available.

### 2.5. Treatment Strategies

The preferred therapy for local CM is surgical excision combined with brachy-, cryo-, chemo- or immunotherapy [24,95] (reviewed in detail by Brouwer et al. [27]). Because of the high recurrence rate of up to 66% [51] after surgical excision in combination with adjuvant therapy, there is a need for new and more successful treatment options. For metastatic disease, there is currently no standardized therapy for CM [20]. Due to missing standards, therapy is often adjusted to cutaneous melanoma. The European guideline for melanoma does not even mention CM separately [96], which does not simplify the treatment of affected patients. Depending on their localization, singular metastases can be surgically removed or irradiated. Until a few years ago, there were no curative therapies for broadly metastasized stages of CM. This situation has improved with the introduction of molecular inhibitors or immunomodulatory therapies. However, only case reports or in vitro studies have been published (compare Table 2).

A wide range of new therapeutic approaches have already made it into clinical trials. Already today, patients with dermal melanoma from AJCC 2017 stage IIIA-D are offered an anti-PD1 therapy, or from stage III A–D with a *BRAF* V600E or V600K mutation are offered a BRAF and MEK inhibitor, as this prolongs the recurrence-free survival, according to the melanoma guideline [97,98,99]. Similar is the approach in patients with CM with or without *BRAF* mutations or advanced disease, although there are no studies on this. Novel treatment options are summarized in Table 2.

**Table 2:** Summary of available molecular inhibitors, immune checkpoint inhibitors and DC vaccination therapeutics, that have been tested in cutaneous and/or CM.

Molecular inhibitors	**Target**	**Commercial Name**	**Tested in**	
			**Cutaneous Melanoma**	**Conjunctival Melanoma**
	BRAF	Vemurafenib	yes [64,100,101,102]	yes (5 patients † + 3 human CM cell lines) [102,103,104,105,106,107]
		Dabrafenib	yes [108,109]	yes (2 patients †) [40,110,111]
		Encorafenib	yes [112]	no
	MEK	Cobimetinib	yes [64,100]	yes (1 patient †) [106]
		Trametinib	yes [108,109]	yes (1 patient † + 3 human CM cell lines) [102,110]
		Binimetinib	yes [112]	yes (only in 3 human CM cell lines) [113]
		Selumetinib	yes [114]	yes (only in 3 human CM cell lines) [102]
	PI3K	Dactolisib	yes [115]	yes (only in 3 human CM cell lines) [102]
		Pictilisib	yes [116]	yes (only in 3 human CM cell lines) [102]
	mTOR	Dactolisib	yes [115]	yes (only in 3 human CM cell lines) [102]
	AKT	MK-2206	yes [117]	yes (only in 3 human CM cell lines) [63]
	MEK	Binimetinib	yes [112]	yes (only in 3 human CM cell lines) [63]
	KIT	Imatinib	yes [118,119,120]	no
	CDK4/6	Ribociclib	yes [121]	no
	ERK1/2	Ulixertinib	yes [122]	no
Immune checkpoint inhibitors	CTLA-4	Ipilimumab	yes [123,124,125]	yes (7 patients ‡) [126,127,128]
	PD-1	Nivolumab	yes [125,129]	yes (4 patients ‡) [126,128]
		Pembrolizumab	yes [130]	yes (7 patients ‡) [103,126,128,131]
DC vaccination		Sipuleucel-T	Clinical trial still ongoing	

† 1 patient received combination therapy of dabrafenib + trametinib, 1 patient received vemurafenib + cobimetinib. ‡ 3 patients received combination therapy ipilimumab + pembrolizumab, 1 patient received ipilimumab + nivolumab.

#### 2.5.1. Molecular Inhibitors

One possibility for new therapy is the treatment with targeted molecular inhibitors. The MAPK and PI3K/AKT/mTOR pathways provide molecular targets [59,65]. Both pathways are highly activated due to mutations in the *BRAF*, *NRAS*, *NF1*, and *KIT* genes as mentioned above. In case reports, BRAF inhibitors in combination with or without MEK inhibitors have shown good therapeutic effects, up to complete response, with acceptable side effects [103,106].

Cao et al. showed that inhibition of both BRAF and MEK act synergistically, at least in three CM cell lines (CRMM1, CM2005 and CRMM2) and was more effective than BRAF inhibition alone [63]. Similar results have been observed in cutaneous melanoma with *BRAF* and *NRAS* mutants, reviewed in [132].

Unfortunately, studies using KIT inhibition with imatinib do not exist in CM [20], probably because *KIT* mutations were rarely detected in CM (see Section 2.3). However, patients with metastatic mucosal melanoma benefit from therapy with imatinib [118,119].

#### 2.5.2. Immune Checkpoint Inhibitors

Further therapeutic options comprise immune checkpoint inhibitors. These therapeutic approaches have recently been applied to advanced or metastatic stages but also in patients with CM lacking *BRAF*- or *KIT* mutants.

Immune checkpoint inhibitors interfere with T-cell function: As mentioned above, DCs are crucial in initiating strong immune responses or inducing tolerance, with both ways being strictly regulated: Induction of tolerance is under the control of immune checkpoint pathways, like cytotoxic T-lymphocyte-associated protein 4 (CTLA-4) or programmed cell death protein-1 (PD-1) [133] and immune stimulation by signals like pathogen-associated molecular patterns (PAMPs) [134]. Therefore, when DCs express CTLA-4 or PD-1, T-cells are inhibited or differentiate into regulatory T-cells [133]. Tumor cells exploit this mechanism by also expressing PD-1 or CTLA-4 to generate an immune-tolerant environment. Immune checkpoint inhibitors (for example ipilimumab, nivolumab and pembrolizumab) are monoclonal antibodies, which target the ligands of these inhibitory T-cell receptors [135]. When the CTLA-4 ligands CD80 and CD86 or the PD-1 ligand PDL-1 are expressed by tumor cells, they effectively inhibit activation of cytotoxic T-cells and foster escape from recognition and elimination by the immune system [65,136,137,138,139]. Immune checkpoint inhibitors stop this inhibitory effect by binding to the regulatory receptors on T-cells, which leads to activation of the immune system against tumor cells [140]. PD-1 is further expressed on natural killer cells, B-cells and monocytes [112,141,142,143]. In CM, PD-1 inhibitor nivolumab induced a complete response to metastatic disease in four patients up to 36 months of follow up [126]. Another PD-1 inhibitor, pembrolizumab, showed responses with mixed results in other case reports [103,126,128].

The CTLA-4 inhibitor ipilimumab is an approved therapy option for metastatic cutaneous melanoma [20]. In a case report using ipilimumab, there was no recurrence of primary or metastatic CM at 16 months of follow-up [127].

Furthermore, in case of insufficient response different immune checkpoint inhibitors can be combined (like a PD-1 inhibitor together with a CTLA-4 inhibitor), however, at the cost of increased side effects including diarrhea, fatigue, pruritus or nausea [128,144,145,146,147]. Ocular side effects are rare (1.3%) [148] and include dry eyes, panuveitis or orbital inflammation [149,150].

Initial clinical studies showed positive effects of therapeutic inhibition of ERK1/2, the PI3K-AKT-mTOR pathway and CDK4/6 in cutaneous melanoma [20]. In the long term, these may also be valid options for the treatment of metastatic CM patients.

Although initial studies have yielded promising results, only case reports of immune checkpoint inhibitors in CM are currently available and no larger, controlled trials have yet been conducted.

#### 2.5.3. DC Vaccination

DC vaccination is another hot topic in oncology and several studies have been performed to understand the mechanisms behind this approach and clinical trials were performed or are ongoing, summarized in [151], although not in CM yet.

DCs are able to initiate adaptive immune responses [152] and activate memory and naïve B- and T- cells as well as cells of the innate immunity including natural killer-cells [153]. To not solely rely on endogenous and in the tumor context often immune-incompetent DCs, there is a need of applying functional DCs as part of antigen-based cancer vaccines [154].

DC vaccination entails the adoptive transfer of autologous, activated, or antigen-loaded DCs into cancer patients. The DCs are isolated or generated in vitro and manipulated before reinfusion [151]. This has already been performed in clinical trials for melanoma and other cancer types [155,156]; however, for melanoma, a clinically approved DC vaccination does not exist so far. Still, the studies showed that DC vaccination is safe and induces antitumor responses [151]. Indeed, for prostate cancer a DC vaccination made it to clinical implementation: Sipuleucel-T (Provenge) is the first FDA approved therapeutic cancer vaccine for metastatic prostate cancer and it prolongs the median survival by 4.1 months [157].

The overall patient response rate using DC vaccination is up to 15% [158,159], which is a significant success because very often these patients suffer from end-stage cancers. López et al. showed increased survival of patients with melanoma after vaccination with DCs loaded with melanoma cell lysates [160]. In this study, 43 stage IV and seven stages III patients were vaccinated with DCs four times [160]. The stage IV patients had an overall survival of 15 months and the stage III patients had a median tumor free period of 48-month follow-up [160].

## 3. The Lymphatic Vasculature in Immune Cell Migration and Tumor Development

**Figure 2:**
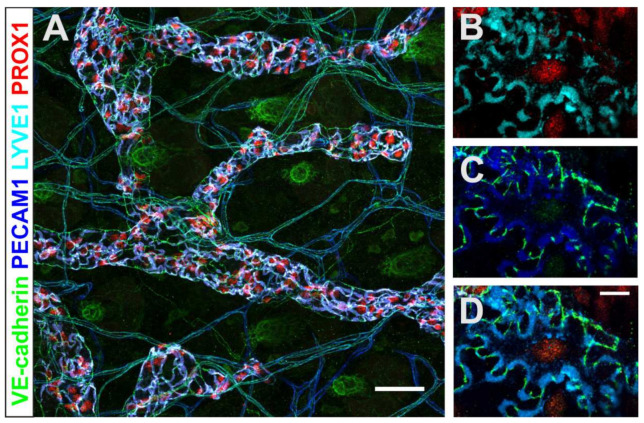
Lymphatic capillaries in mouse dermis. Such high-resolution images of the conjunctiva are not yet available. (**A**) Maximum intensity projection of wholemount stained mouse dermal lymphatic capillaries. The combination of endothelial-specific (VE-cadherin, PECAM-1) lymph vessel specific (PROX1) and capillary (LYVE1) markers unambiguously identifies lymphatic capillaries. (**B–D**) Higher magnification depicts the prototypic oak-leaf shape of capillary LECs and the button junctions formed by VE-cadherin, which alternates with LYVE1 and PECAM-1. The colors of the depicted epitopes are indicated on the left. Scale bars (**A**) 50 µm, (**D**) 10 µm.

Lymphatic vessels play a central role in the maintenance of tissue fluid balance, dietary lipid absorption and the trafficking of immune cells [161]. In healthy tissue, blind ending initial or capillary vessels of the mature lymph vessel system take up interstitial fluids, solutes and extravasated immune cells and transport this complex mixture “the lymph” via collecting vessels to the draining lymph nodes [162,163]. For efficient uptake, capillary lymphatic endothelial cells (capLECs) are endowed with specialized button junctions where focal accumulations of endothelial adhesion and tight junction molecules alternate with long intermittent membrane stretches rich in PECAM-1 and the hyaluronan receptor LYVE-1 [161,164], leading to a characteristic oak leaf shape (Figure 2).

From capillaries, the lymph is channeled via pre-collectors into collecting lymphatic vessels, which carry it to the regional lymph nodes and after exiting through efferent lymphatics via the left and right lymphatic duct to the venous circulation.

Elegant cannulation studies, which analyzed the composition of lymph by tracing immune cells, labeled with photo-convertible fluorescent proteins, identified T-cells as major cargo of afferent lymphatics followed by antigen presenting DCs and few B-cells [165,166]. Performing continuous immune surveillance for cognate antigen, these populations enter afferent lymphatics and rapidly stimulate recall immune responses in the draining regional lymph nodes. Interestingly, the overwhelming majority of recirculating T-cells in the lymph are CD4+, of which 25% are comprised of FOXP3+ regulatory T-cells. The high percentage of Tregs in lymph provides solid evidence for the role of lymphatic vessels in immunoregulatory functions [166]. In the steady state, few DCs that populate the lymph clearly also contribute to the maintenance of peripheral and oral tolerance [167,168]. DC numbers massively rise during inflammation, when DCs present immune stimulatory antigens in the regional nodes [169]. In all these scenarios, the major chemokine produced by lymphatic capillaries is CCL21, guiding vessel entry of CCR7+ T-cells and DCs [170,171,172,173].

Developmentally, already the earliest lymphatic progenitors express the transcription factor PROX1 and appear at the dorsal roof of the cardinal vein [174]. PROX1 is essential for the differentiation and maintenance of LECs. Through further migration and sprouting, LECs form a primitive lymphatic plexus, in a process that is regulated and dependent on the vascular endothelial growth factor receptor-3 (VEGFR3). Two cognate growth factors VEGF-C and -D trigger VEGFR3 signaling-mediated responses in LECs, but developmental regulation overwhelmingly depends on VEGF-C [163,175].

Following postnatal maturation, the lymphatic vessel system acquires a quiescent state. LEC quiescence may also be revoked by lymphangiogenic, physiologic, but mainly pathologic stimuli, including tumor development, but also wound healing, obesity, or inflammatory scenarios like myocardial infarction, inflammatory bowel disease, or corneal transplantation [176,177,178]. In inflammatory scenarios, the action of lymphatic vessels is Janus-faced. While they provide easy access for pro-inflammatory antigen presenting cells to the regional lymph nodes, at the same time they contribute to antigen clearance and facilitate the trafficking of immunomodulatory cells, including the aforementioned Tregs, and thereby can contribute to the efficient resolution of inflammation. Many tumor cells, but also tumor-associated inflammatory cells are rich sources of VEGF-C and VEGF-D, causing the development of extensive peri-tumoral but also tumor-invading lymph vessels that may serve as conduits for metastasis dissemination [179,180]. CD146, a cell adhesion molecule, is involved in driving melanoma progression and metastasis, particularly in vascular and lymphatic metastasis via VEGF-R2 [181]. Tumor associated lymphangiogenesis is generally associated with poor prognosis [182,183], however, at the same time tumor lymph vessels may provide benefits for the generation of an anti-tumor immune response during checkpoint inhibition immune therapy [184,185,186].

The conjunctiva contains lymphatic vessels, which lately have been shown ex vivo using OCT signaling in porcine eyes [187] or in vivo in mice and rats [188]. In human fetal samples, the conjunctiva shows lymphatic vessels as early as week 17 of gestation [189]. Even in adults, the lymphatic status of ocular tissues is actively regulated by pro- and angiogenic factors [190].

Regarding CM, the presence of lymphatic vessels has been demonstrated in a CM mouse model [191], as well as in human CM samples [192,193]. In patients, lymphangiogenesis was associated with an increased risk of recurrence, metastasis and tumor-related death [194]. Tumor samples expressed VECF-C, -D and -R3, as well as CXCL12, CCL21 and CXCR4 with the highest lymphatic vessel density at the invasive tumor edges [195]. In CM, the lymphangiogenic stimulus is triggered very early, even before the invasive phenotype of the tumor appears [192,194].

The interaction of DCs and tumor draining lymphatic vessels but also tumor-induced changes in lymph nodes have been studied poorly in CM. Tumor draining lymph nodes often present an altered immunologic microenvironment compared to non-tumor-associated lymph nodes. An increased frequency of regulatory T-cells in tumor-draining lymph nodes correlates with a worse prognosis for cutaneous melanoma patients. [196]. This is another potential therapeutic option to not only strengthen DCs locally in their antitumor response but also to promote their pathway to the lymph nodes, where the immune system is activated against the tumor.

## 4. Dendritic Cell Function in General, in the Conjunctiva and in (Conjunctival) Melanoma

DCs are professional antigen presenting cells, which capture, process and present antigens to T-cells [197] after reaching the draining lymph nodes via the lymphatic vessels as described in Section 3. DCs link the innate and adaptive immune systems and express a high level of major histocompatibility complex (MHC) [198]. DCs develop from DC precursor cells in the bone marrow [199] and are classified into different subsets. The classification of these subsets, based on the expression of different cell surface markers [199] (Table 3 and Table 4), is listed in detail below.

### 4.1. Lymphoid Conventional Dendritic Cells

The main function of conventional DCs (cDCs) is antigen presentation [204]. Their precursor cells leave the bone marrow and migrate to peripheral secondary lymphoid organs like the spleen and lymph nodes [204]. cDCs are subdivided into two subsets, cDC_1_ and cDC_2_.

#### 4.1.1. cDC_1_

In mice, lymphoid cDC_1_ express toll-like receptor 2, 3, 4, 9, 11, 12 and 13 (also called pathogen recognition receptors PRRs) and recognize pathogen associated molecular patterns (PAMPs) [205,206]. cDC_1_ are the main producers of interleukin 12 (IL-12), interferon α (IFN-α) and IFN-λ and also express IL-6 and tumor necrosis factor α (TNF-α) when they are activated by PRRs [199,207]. cDC_1_ in mice recognize exogenous soluble and cell-associated antigens which they present to CD8+ T-cells on MHCI [208,209]. They recognize apoptotic and necrotic cells, via Clec9A and present their antigens to cytotoxic CD8+ T-cells [210,211,212].

In the tumor context, cDC_1_ are also specialized to take up material from dead tumor cells and transport it to tumor-draining lymph nodes where they present it to anti-tumor CD8+ T-cells [213,214].

Although in skin melanoma, the amount of cDC_1_ cells is low, e.g., in tg(Grm1)EPv mice, the amount of cDC_1_ positive cells in skin melanomas was less than 1% of the CD45+ cells, compared to more than 2% cDC_2_ and about 8% Langerhans cells [215], cDC_1_ still had important anti-tumoral effects. Intratumoral cDC_1_ can attract T-cells [216], stimulate and expand CD8+ T-cells [217] and secrete IL-12 [218]. In Batf3^−/−^ mice, cDC_1_ are missing and this leads to a failure to reject immunogenic tumors and a worse response to immune checkpoint therapies [214,216,217,219].

Human cDC_1_ express TLR1, 3, 6, 8 and 10 [200,220,221]. As in mice, human cDC_1_ are the main producers of type 3 interferon and cross-present antigens to cytotoxic T-cells [222]. In contrast to mice, human cDC_1_ are able to stimulate allogeneic or autologous T-helper cells [223].

#### 4.1.2. cDC_2_

Lymphoid cDC_2_ in mice present pathogens on MHCII molecules [200,224]. cDC_2_ in the spleen detect pathogens within the cytoplasm and express TLR 1, 2, 4, 5, 6, 7, 8, and 9 [205,206]. Induced by activated TLR, cDC_2_ express proinflammatory cytokines like TNF-α, IL-6 and low levels of IL-10 and play an important role in chemo attraction [199,225].

cDC_2_ can be subdivided in ESAM^hi^ (80% of cDC_2_), which are more efficient in Th_2_ polarization and in ESAM^lo^ [226,227] cDC_2_, which prime Th_1_ cells more efficiently [199,227].

Human cDC_2_ are among others CD11c^hi^ SIRPα^+^ and can be subdivided into CD5^hi^ and CD5^lo^ cDC_2_ [200,228]. CD5^hi^ DCs showed better migration to the lymph node and induce more potently T-cell proliferation, compared to CD5^lo^ cDC_2_ [229], indicating contrasting roles in tolerance and immunity.

In the peripheral blood of melanoma patients, no significant difference was seen regarding disease stage and CD11b+ DCs [230]. In a transgenic tg(Grm1)EPv melanoma mouse strain, the amount of cDC_2_ decreased in the skin melanoma compared to the tumor free tissue over time, whereas no difference was seen for Langerhans cells (LCs) and cDC_1_ [215]. Binnewies et al. showed that in mice and humans, that CD11b+ cDC2 and BDCA-1+ cDC_2_ are important to induce a protective antitumor CD4^+^ T-cell immune response and may act as a target of T-reg suppression [224].

### 4.2. Migratory Dendritic Cells (mDCs)

Tissue migratory or non-lymphoid DCs in mice can be subdivided into three main subsets: **CD103++ CD11b- mDCs**, **CD103- CD11b+ mDCs** and **CD103++ CD11b+** (intestinal DCs) [200]. Further expressed markers are listed in Table 3. The CD103+ CD11b- subset has the same origin and function as cDC_1_ and are found mainly in connective tissues [231]. The intestinal DCs are found in the Peyer’s patches [232]. CD103- CD11b+ DCs consists of a mixture of tissue DCs and macrophages [200]. Tissue-migratory DCs play a role in tissue immune responses and migrate via CCR7 to T-cell areas in the lymph node [171,200,233]. In human, non-lymphoid migratory DCs are subdivided in two subsets: CD1a- CD14+ CD163- and CD1a+ CD14- SIRPα+, which are located in the dermis [200].

In mouse skin melanoma models, CD103+ CD11b+ cells have been shown to be the only antigen presenting cells, transporting tumor antigen to the lymph nodes and subsequently priming CD8+ T-cells, although they represent only a minor population of DCs infiltrating the tumor [214]. They were further necessary to promote an anti-tumoral effect upon blockade of PDL-1 and could be systemically expanded by Flt3L with a tumor protective effect [214].

### 4.3. Langerhans Cells (LCs)

LCs are found in the epidermal layer of the skin, in the epithelium of the cornea, buccal, gingival and genital mucosae and lead to regulatory T-cell proliferation in a steady state, but limit the activation in an inflamed state [234,235].

Human LCs are among others LANGERIN+ [200]. In humans, LCs are producers of IL-15 [236]. They are able to present antigens to cytotoxic CD8^+^ T-cells, to Th_17_ and Th_2__2_ cells [236,237,238]. In different mucosal tissues, there were highly site dependent differences in the amount of LCs, when comparing the amount of LC in the conjunctiva, oral mucosa, anus and penis [239].

### 4.4. Plasmacytoid Dendritic Cells (pDCs)

pDCs are the major producers of type 1 interferons [199,204] and have anti-proliferative and antiviral functions [240]. pDCs develop in the bone marrow and afterward circulate in the blood and migrate to peripheral organs [202,241].

pDCs in mice are CD317+ CD209++ Ly6c++ [200,202]. TLR7, TLR9 and TLR12 are highly expressed by pDCs [205]. pDCs upregulate MHC II and produce type I (IFN-α, IFN-β) and III interferons (IFN-λ), which affect cDCs, NK cells, CD4^+^ T-helper cells and cytotoxic T-cells to lead to an anti-viral immune response [202,242].

Human pDCs, like murine pDCs, respond to viral infections and produce type 1 interferon in response to TLR7 and TLR9 and type 3 interferons in response to TLR9 [243,244]. The presence of pDCs in tumors is correlated with a worse outcome because they promote the expansion of T-reg populations, which inhibit the anti-tumor immunity [243]. Low pDCs levels in the peripheral blood of melanoma patients were correlated with advanced or active melanoma [230].

Others showed that human pDCs are more efficient in attracting cytotoxic CD8+ T-cells than cDC_2_ in melanoma patients receiving DC vaccination [245]. Interfering with pDC function has led to therapeutic innovations in skin cancers like basal cell carcinoma. The TLR7 ligand *Imiquimod* has been approved for local treatment and promotes pDCs and induces cytotoxicity [246].

### 4.5. Monocyte Derived Inflammatory Dendritic Cells (moDCs)

moDCs originate from monocytes and appear in inflammation, cancer, or infection [203,237,247]. moDCs act at the site of inflammation and stimulate CD4^+^ and CD8^+^ T-cells due to the secretion of IL-1, IL-12, IL-23 and TNF-α [237,248].

### 4.6. Dendritic Cells in Healthy Conjunctiva and during Conjunctival Pathologies

In general, little is known about DC subsets in the conjunctiva per se and in CM. However, a few DC subsets have been described in the healthy conjunctiva: Ohbayashi et al. used confocal microscopy for detecting DCs in the conjunctiva and found a small number of conventional DCs but almost no plasmacytoid DCs (pDCs) [249]. Langerhans cells (LCs) were also absent from the conjunctival epithelium [249]. In contrast, Jamali et al. (2020) reported a population of pDCs in the bulbar conjunctiva [250]. In pigmented conjunctival lesions, DCs have been detected using in vivo confocal microscopy [251,252]. A subset differentiation was not possible using this technique. Older studies identified LCs in the conjunctiva at a density of up to 300 cells/mm^2^ [253] and with a decline in the LC density corresponding to the advancing age of tissue donors. Most LCs were found in the palpebral conjunctiva [254]. Khandewal et al. could show in the mouse that CD103+ as well as CD11b+ DC are present in the healthy conjunctiva and induce allergic responses [255] via CCR7 [256].

DCs play also an important role in the development of autoimmune diseases of the ocular surface like dry eye disease. It was shown that Thrombospondin-1 (TSP-1) is expressed on DCs of the ocular surface [257].

Additionally, in infectious diseases, DCs play a critical role by presenting pathogenic antigens to effector T-cells. For the conjunctiva, it was recently shown that goblet cells, which provide the mucin layer of the tear film, are in close contact with conjunctival DC. Contreras-Ruiz et al. could show that, on one hand, goblet cells control the activation status of conjunctival DC by expression of TGF-β2 and TSP-1 inducing a tolerogenic phenotype [13]. On the other hand, they could show that goblet cells responding to TLR5 engagement activate conjunctival DC, which subsequently extend their intra-epithelial dendrites and upregulate MHC class II [258].

In pterygium, a benign conjunctival tumor that centripetally invades the clear cornea, laser scanning confocal microscopy revealed in patients that in both the clear cornea, as well as in the pterygium, the amount of DCs was significantly increased [259]. An Australian study analyzed the predictive value of pterygia for cutaneous melanoma and found significantly more cases of cutaneous melanoma in patients with pterygium compared to the control cohort [259].

In malignant CM, Wolf et al. recently characterized the tumor microenvironment of CM in more detail. Next to natural killer T-cells (NKT), B-cells and mast cells, they found an enrichment of pDC and activated DCs in CM compared to healthy conjunctiva [260]. For pDCs it is known that they originally contribute a strong IFNγ response within the innate and adaptive immune response. In tumorigenesis, in contrast, pDCs produce less IFNγ and contribute to an immunosuppressive tumor microenvironment (TME), as summarized elsewhere [261].

In the field of skin melanoma, more is known about the DC subtypes. Here three major subsets of CD11c^+^ MHCII^+^ DCs have been identified in the tumor microenvironment: cDC_1_, cDC_2_ and moDCs [262,263,264].

In several studies of human melanomas, a low number of intratumoral DCs have been associated with a worse prognosis [265,266].

Wculek et al. reviewed that tumor-associated DCs, for example, in ovarian cancer, cutaneous melanoma, gastric cancer and lung carcinoma have a reduced function in differentiation, activation and stimulation of immune response [216,243,267,268,269].

## 5. Summary

CM is a very rare tumor with a high rate of mortality and local recurrence. Despite some similarities with cutaneous melanoma, CM is a mucosal melanoma and only very little is known about immunological responses or DC subsets in CM. Promising new therapeutic options are targeted molecular inhibitors, immune checkpoint inhibitors and DC-immunotherapy. Although more and more knowledge about DCs and their subtypes is becoming known and single studies have shown effects of different DC subtypes in several diseases of the ocular surface, unfortunately very little is known about the subtypes in CM so far. Whether individual subtypes are important and similar to other cancer types, should be shown by future studies to exploit possible therapeutic effects.

Therefore, much remains to be learned about the composition and function of DCs and their subsets in CM to establish new therapy options.

## Figures and Tables

**Table 1:** Similarities and differences between conjunctival, cutaneous and other mucosal melanomas.

	Similarities of Cutaneous, Mucosal and Conjunctival Melanoma:	Differences Between Cutaneous, Mucosal and Conjunctival Melanoma:
Origin, embryology and anatomical characteristics	- All arise from melanocytes [6]. - Conjunctiva, mucosa of the head, e.g., oral or nasal mucosa and skin originate from the surface ectoderm [7]. - All three contain immune cell patches with T- or B-cells: the so called conjunctiva-associated lymphoid tissue (CALT) [8], the mucosal associated lymphoid tissue (MALT) or the skin-associated lymphoid tissue (SALT) [9].	- CM has an average thickness of only 33 µm [10], compared to the dermal epithelium thickness of an average of 2 mm and mucosal thickness, e.g., oral mucosa 0.1–0.5 mm. - Conjunctiva and mucosa contain several anatomical particularities different from the skin [11], e.g., goblet cells, which produce mucin [12], express transforming growth factor β2 (TGF-β2) and are able to induce tolerogenic DCs [13,14]. Loss of goblet cells correlates with more severe conjunctival diseases, like dry eye syndrome [15]. - The skin is keratinized, whereas conjunctiva and mucosa are non-keratinized.
Incidence		- Highest incidence (per year) in cutaneous melanoma (19.7/100,000) [16], compared to 0.2–0.8/1,000,000) in CM [17], and 1.5–2.8/1,000,000 cases in mucosal melanoma [18].
Risk factors		- UV radiation is a clear risk factor in cutaneous melanoma, whereas in CM the role of UV radiation is not completely understood [19] and in mucosal melanoma UV radiation is not of importance.
Mutations	- Cutaneous melanoma and CM show similarly high mutation rates in the *BRAF*, *NRAS*, *TERT* and *KIT* genes [20].	- *TERT* mutations are very rare in mucosal melanoma, whereas *KIT* mutations are the most frequently altered oncogene in mucosal melanomas [21] (different from cutaneous or CM).
Metastasis	- All metastasize via lymphatic and hematogenous spread [17].	

**Table 3:** Mouse DC subsets and their expressed surface markers [200,201,202,203].

	Conventional DC		Migratory DC			Langerhans Cells	pDC	moDC
	cDC_1_	cDC_2_	CD103+ CD11b-	CD103- CD11b+	CD103+ CD11b+			
Expressed surface markers	CD11c+++ CD45R- MHC II++ CD8α+ CD11b+ Sirpα+ CD24++ CD26+ XCR1+ CD205++	CD11c+++ CD45R+ MHC II+ CD8α- CD11b+ CD24+ CD26+ XCR1- CD172α++ CD205+	CD11c+ CD103++ CD11b- MHC II++ CD209 (dc-sign)- CD172α- Ly6c-	CD11c+ CD103- CD11b+ MHC II++ CD11c++ CD172α++	CD11c+ CD103++ CD11b+ MHC II++ CD11c++ CD209 (dc-sign)+ Ly6c- CD172α-	CD45+ CD11c++ MHC II++ CD11b+ CD8- CD24++ CD205++ CX_3_CR1+ CD172α+ F4/80+	CD11c+ CD45RA+++ CD45R+++ CD317+ MHC II+ CD172α+ CD11b- CD209 (dc-sign)++ Ly6c++	MHC II+ CD11b+ CD11c+ Ly6c+ F4/80+ FcεRI+ CD209 (dc-sign)+ CD64+

**Table 4:** Human DC subsets and their expressed surface marker [194,196,197,198].

	Conventional DC		Migratory DC		Langerhans Cells	pDC	moDC
	cDC_1_	cDC_2_	CD1a- CD14+	CD1a+ CD14-			
Expressed surface markers	HLA-DR+ CD11c+ CD11b- SIRPα- CD141+ CLEC9A+ XCR1+	HLA-DR+ CD11c+++ CD123- SIRPα+ CD1c+ CLEC9A- BDCA1+	Lin- HLA-DR+ CD11c+ CD1a- CD14+ BDCA1+ LANGERIN- CD163- EpCAM-	Lin- HLA-DR+ CD11c+ CD1a+ CD14- BDCA1+ LANGERIN- EpCAM- SIRPα+	Lin- HLA-DR+ CD11c+ CD1a+ CD14- BDCA1+ LANGERIN+ E-CADH+ SIRPα+	Lin- BDCA2+ BDCA4+ HLA-DR+ MHC II+ CD1a-	HLA-DR+ CD11c+ BDCA1+ CD1a+ FcεRI+ CD11b+ CD172α+ CD206+ CD14+
